# Supplementary figures and images for: Physiological and Comparative Transcriptome Analyses of the High-Tillering Mutant mtn1 Reveal Regulatory Mechanisms in the Tillering of Centipedegrass (Eremochloa ophiuroides (Munro) Hack.)
Source: Int J Mol Sci. 2022 Sep 30;23(19):11580. doi: 10.3390/ijms231911580 (PMC9569434; doi:10.3390/ijms231911580)

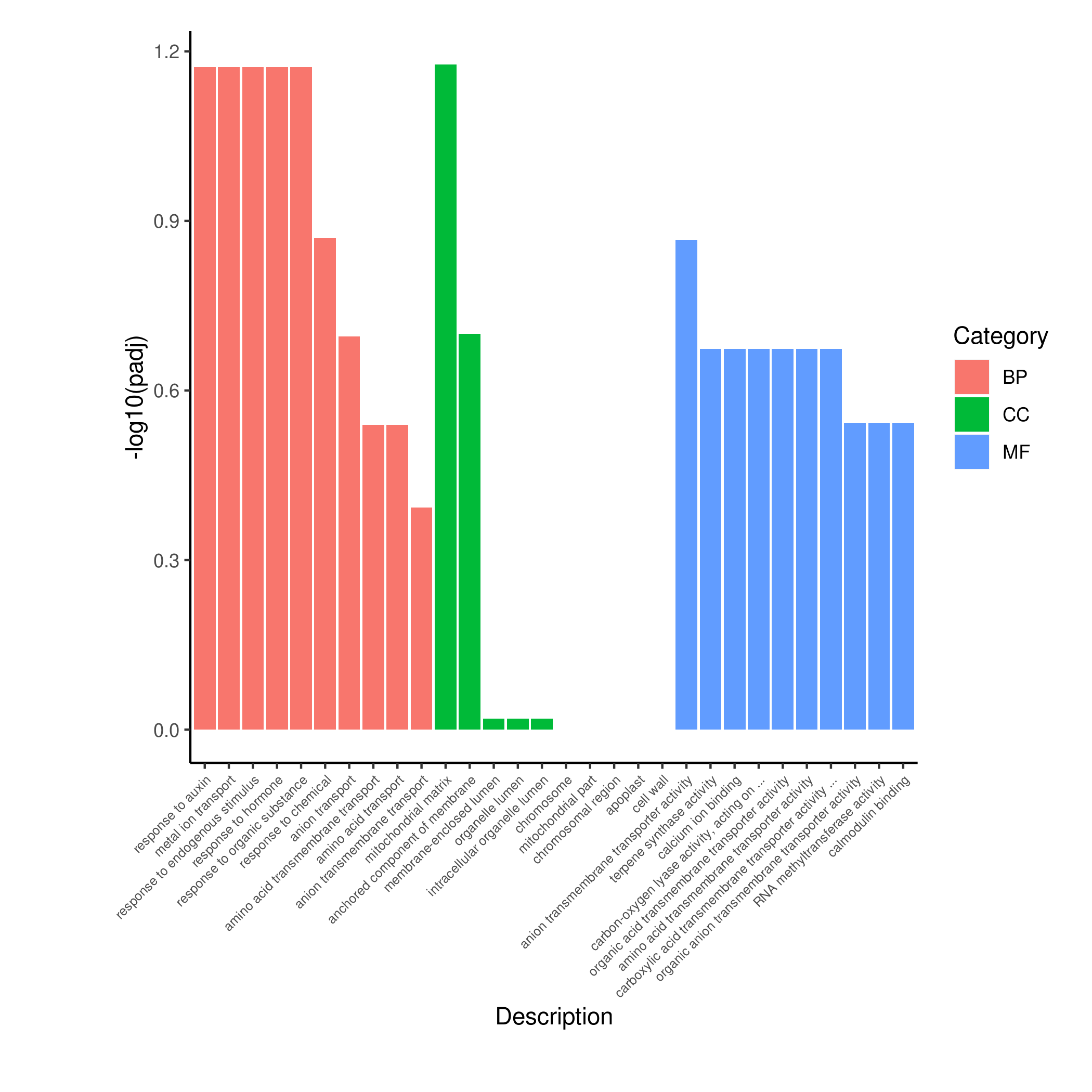

Supplement: Supplementary file 1 [file ijms-23-11580-s001.zip › Figure S1.png]

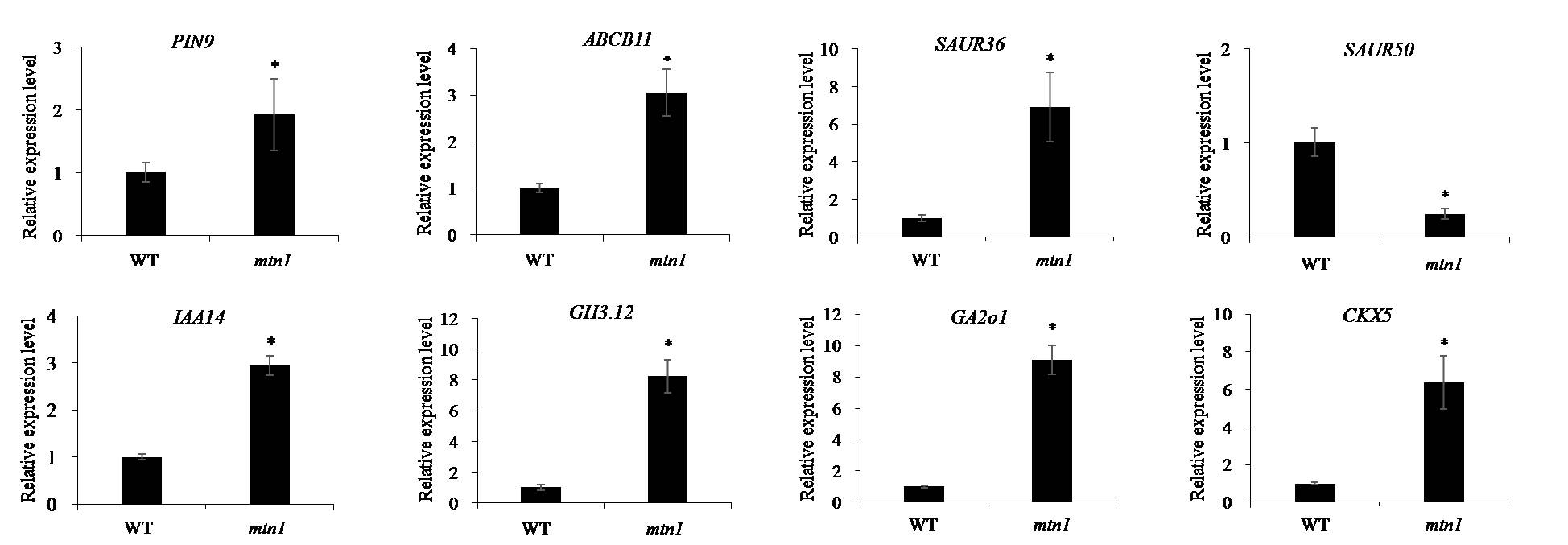

Supplement: Supplementary file 1 [file ijms-23-11580-s001.zip › Figure S2.jpg]

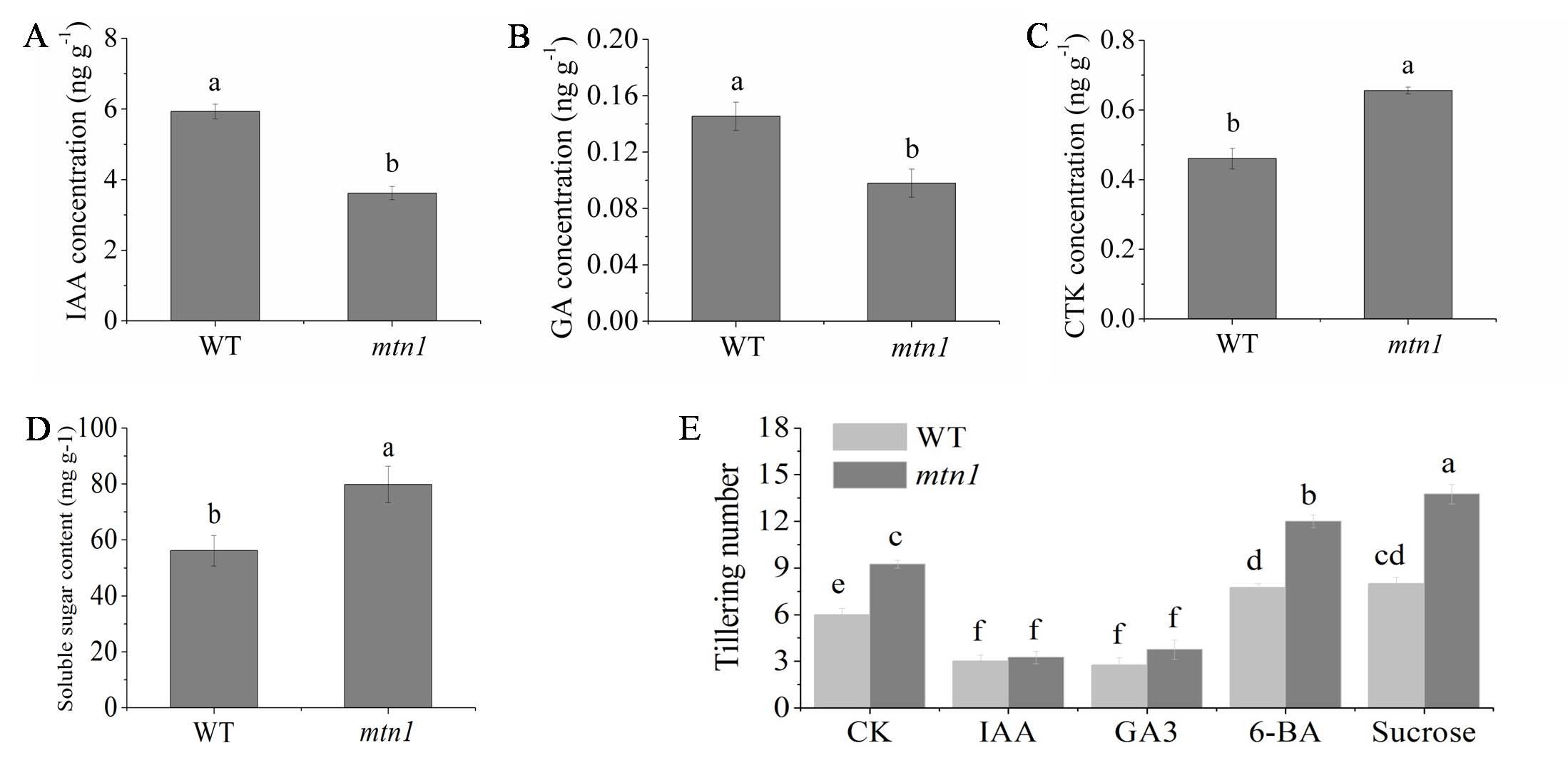

Supplement: Supplementary file 1 [file ijms-23-11580-s001.zip › Figure S3.jpg]
